# Intraoperative Blood Loss Independently Predicts Survival and Recurrence after Resection of Colorectal Cancer Liver Metastasis

**DOI:** 10.1371/journal.pone.0076125

**Published:** 2013-10-01

**Authors:** Wu Jiang, Yu-Jing Fang, Xiao-Jun Wu, Fu-Long Wang, Zhen-Hai Lu, Rong-Xin Zhang, Pei-Rong Ding, Wen-Hua Fan, Zhi-Zhong Pan, De-Sen Wan

**Affiliations:** 1 Department of Colorectal Surgery, Sun Yat-sen University Cancer Center, Guangzhou, China; 2 Department of Experimental Research, Sun Yat-sen University Cancer Center, Guangzhou, China; 3 State Key Laboratory of Oncology in South China, Sun Yat-sen University Cancer Center, Guangzhou, China; University Hospital Carl Gustav Carus Dresden, Germany

## Abstract

**Background:**

Although numerous prognostic factors have been reported for colorectal cancer liver metastasis (CRLM), few studies have reported intraoperative blood loss (IBL) effects on clinical outcome after CRLM resection.

**Methods:**

We retrospectively evaluated the clinical and histopathological characteristics of 139 patients who underwent liver resection for CRLM. The IBL cutoff volume was calculated using receiver operating characteristic curves. Overall survival (OS) and recurrence free survival (RFS) were assessed using the Kaplan–Meier and Cox regression methods.

**Results:**

All patients underwent curative resection. The median follow up period was 25.0 months (range, 2.1–88.8). Body mass index (BMI) and CRLM number and tumor size were associated with increased IBL. BMI (*P*=0.01; 95% CI = 1.3–8.5) and IBL (*P*<0.01; 95% CI = 1.6–12.5) were independent OSOs predictors. Five factors, including IBL (*P*=0.02; 95% CI = 1.1–4.1), were significantly related to RFS via multivariate Cox regression analysis. In addition, OSOs and RFS significantly decreased with increasing IBL volumes. The 5-year OSOs of patients with IBL≤250, 250–500, and >500mL were 71%, 33%, and 0%, respectively (*P*<0.01). RFS of patients within three IBL volumes at the end of the first year were 67%, 38%, and 18%, respectively (*P*<0.01).

**Conclusions:**

IBL during CRLM resection is an independent predictor of long term survival and tumor recurrence, and its prognostic value was confirmed by a dose–response relationship.

## Introduction

The liver is the most common target organ of distant colorectal cancer metastasis. 20%-25% percentage points newly diagnosed patients present with synchronous liver metastases and approximately 50% patients develop metachronous liver metastases after radical resection for primary colorectal cancer [1,2]. Untreated patients have a poor survival of only 6–12 months [3,4]; however, chemotherapy plus molecular targeted agents can prolong the median survival between 12 to 24 months [5,6]. Nevertheless, comprehensive management with curative liver resection for colorectal cancer liver metastasis (CRLM) offers a 5-year survival rate of up to 58% [7-9]. Because of the advances in equipment and improvements in surgical techniques, perioperative morbidity and mortality following hepatectomy have significantly declined in recent years; however, excessive intraoperative blood loss (IBL) remains a significant concern for surgeons. Several studies have focused on the relationship between IBL during liver surgery for hepatocellular carcinoma (HCC) and postoperative outcome and reported that IBL was an independent prognostic factor [10], but few studies have been devoted to CRLM. Therefore, in the present study, we evaluated the influence of IBL during liver resection for CRLM on tumor recurrence and long term survival.

## Methods

### Ethics statement

The study was performed following approval by the ethic committee of Sun Yat-sen University Cancer Center. We were replied that it’s not necessary to get signatures of patients’ informed consent form, it’s according to the current Chinese medical regulations, the process of whole study is non-invasive, and without any patients’ benefit hurt. However, each patient has signed the informed consent form before surgical operation. Ethics committees approved this consent procedure.

### Study Population and Clinicopathological Factors

A total of 163 consecutive patients, who underwent liver resection for CRLM at Sun Yat-sen University Cancer Center (Guangzhou, China) from January 2005 to December 2011, were reviewed. Because the current study focused on long term survival and tumor recurrence after hepatectomy, we excluded 20 patients who underwent R1 or R2 resection for CRLM and 4 patients with uncertain postoperative courses. The remaining 139 patients underwent curative resection as indicated by complete tumor clearance of both primary and metastatic lesions.

Patient demographics as well as clinical, histopathological, and laboratory data were collected. Body mass index (BMI) was obtained for each patient on admission, and complete blood counts, blood chemistry profiles, and serum tumor marker tests were routinely performed within 1 week before surgery. The neutrophil to lymphocyte ratio (NLR) was calculated from the differential count by dividing the absolute neutrophil count by the absolute lymphocyte count, and the ratio between serum levels of low density lipoprotein (LDLC) and high density lipoprotein (HDLC) was determined. Hepatitis B virus (HBV) infection was consist of chronic hepatitis B (presence of HBsAg and HBeAg), inactive carriers (presence of HBsAg, and absence of HBeAg or presence of anti-HBe), and resolved hepatitis B (negativeHBsAg, and the presence of anti-HBc and/or anti-HBs) [11,12]. Pathological findings for primary and metastatic lesions were documented in written reports, and all tumors were pathologically staged according to the American Joint Committee on Cancer (AJCC; 7th edition) staging system.

### Surgery

All patients were recommended for multidisciplinary treatment before initial therapy. Indications for liver resection were as follows: (1) sufficient medical fitness for major laparotomy, as indicated by favorable Child–Pugh status, which usually included only class A patients; (2) radiological evidence of resectable lesions (i.e., complete resection of all liver metastases, regardless of size, number, distribution, or width of resection margin); and (3) at least 30% normal liver parenchymal volume can be preserved as estimated by preoperative computed tomography and/or magnetic resonance imaging.

Palpation and intraoperative ultrasonography were performed for all the patients to identify occult liver metastases and to confirm tumor number, size, and location, particularly in relation to the hepatic and portal veins. Anatomic resection refers to the en bloc removal of the hepatic segments as defined by Couinaud. Nonanatomic resection means surgical excision of the tumor without regard to hepatic anatomy. The type of hepatectomy was determined by the attending surgeons based on tumor number, location and patient status. Low central venous pressure general anesthesia and intermittent Pringles portal occlusion were employed to minimize IBL. Moreover, radiofrequency or microwave ablation was used as a combination therapy in 15 patients to achieve R0 resection because of unfavorable tumor locations. The amount of IBL was calculated in 50-mL increments by adding the contents of the suction containers to the weight of the laparotomy sponges at the end of the surgery. Furthermore, administration of allogeneic red blood cells or blood plasma was considered as a transfusion event.

### Follow up

Patients were followed up every 3 months for the first 2 years, every 6 months for the next 3 years, and once annually thereafter. Data including clinical examinations, blood tests, and radiologic evaluations, were documented. Follow up of patients residing at a distance from the hospital was usually performed by the local referring clinician, and the data were obtained through direct contact with patients or their families by telephone.

Recurrence was defined by clinical, radiological, and/or pathological diagnosis of tumors emerging from previous local or distant origins. Increased serum levels of carcinoembryonic antigen (CEA) without other evidence of recurrence at suspected anatomical sites were not considered as metastases or recurrent diseases.

### Statistical analysis

Data were analyzed using SPSS 16.0 statistical software (SPSS, Inc., Chicago, IL, USA). We selected the cutoff values for IBL, BMI, NLR, LDLC/HDLC, CEA, and CRLM size using receiver operating characteristic (ROC) curve analysis. At each value, the sensitivity and specificity for each outcome under study was plotted to produce ROC curves. The score closest to the point of the maximum sensitivity and specificity was selected as the cutoff value. Clinicopathological predictors of IBL were identified using logistic regression analysis. Overall survival (OS) and recurrence-free survival (RFS) were calculated using the Kaplan–Meier method. Differences between patient groups were compared using the log rank test. All probability (*P*) values were 2-sided and a *P* value <0.05 was considered statistically significant. Moreover, a Cox proportional hazards model (forward) was used for multivariate analysis to identify independent prognostic factors. Only potential predictive factors on univariate analysis (p<0.05) were taken into the Cox model. The hazard ratio (HR) and 95% confidence interval (CI) were used to estimate the role of each independent prognostic factor.

## Results

### Baseline patient characteristics

The patient characteristics are summarized in Table 1. The median age of the 139 patients was 58 years (range, 25–82 years), with 91 (65.5%) males and 48 (34.5%) females. Most (105, 77%) of the patients had BMI<25.3, and only 10% had a medical history of type 2 diabetes mellitus. HBV infection occurred in 42% of the entire patient cohort, including 1 patient with chronic hepatitis B, 13 inactive carriers,and 45 patients withresolved hepatitis B. Primary tumors were in the colon in 78 (56%) patients and in the rectum in 61 (44%). In 76 patients, the primary tumors involved lymph nodes metastasis. Approximately 50% patients had multiple liver metastases with a maximal tumor number of 13.The median CRLM tumor size was 2.5cm (range, 0.3–11.5 cm). Preoperative chemotherapy was administered to 67 (48%) patients. Anatomic hepatectomy was performed in 17 (12%) patients, while othersreceived nonanatomic resection. Moreover, the perioperative morbidity rate was 12% because of hemorrhage, intra-abdominal abscess, wound infection, pleural effusion, bile leak, hepatic failure, intestinal obstruction, and cardiovascular events. There was no perioperative death within 30 days after surgery.

**Table 1:** Patient characteristics.

	**Characteristics**	**N (%)**
**Age^†^**	≤60y	80 (58)
	>60y	59 (42)
**Gender**	Male	91 (65)
	Female	48 (35)
**BMI (kg/m^2^)^∗^**	≤25.3	105 (77)
	>25.3	31 (23)
**DM^∗^**	Yes	14 (10)
	No	124 (90)
**HBV infection**	Yes	59 (42)
	No	80 (58)
**Primary tumor**	Colon	78 (56)
	Rectum	61 (44)
**LN metastasis of primary tumor**	N_0_	43 (36)
	N_1–2_	76 (64)
**Number of CRLM**	Solitary	73 (53)
	Multiple	66 (47)
**Size of CRLM (cm)^∗^**	≤4.5	103 (75)
	>4.5	34 (25)
**Exhepatic metastasis**	Yes	12 (9)
	No	127 (91)
**Preoperative chemotherapy**	Yes	67 (48)
	No	72 (52)
**IBL(mL)**	≤250	87 (63)
	250–500	36 (26)
	>500	16 (12)
**Perioperative transfusion**	Yes	36 (26)
	No	103 (74)
**Periopoerative morbidity**	Yes	17 (12)
	No	122 (88)
**Type of hepatectomy**	Anatomy	17 (12)
	Nonanatomy	122 (88)

† Median age: 58years (range, 25–82 years)

∗ For some characteristics (i.e., BMI, DM, and CRLM size), data were not available for all patients.

### Related Factors for Increasing IBL

**Figure 1:**
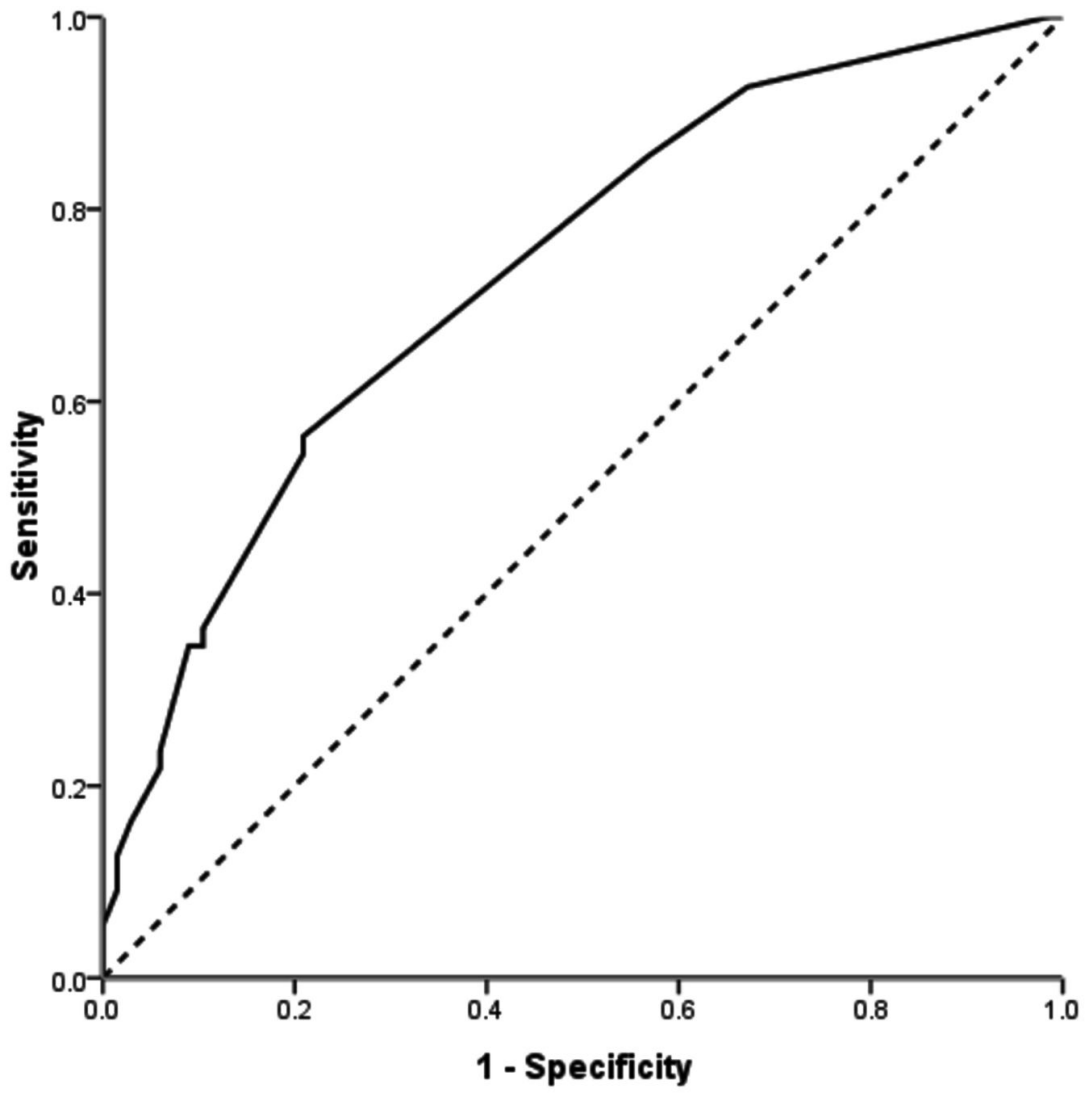
ROC curve to determine the IBL cutoff volume.

The median IBL was 200mL (range, 50–4000mL), and 36 (26%) patients received perioperative blood transfusions. An IBL volume of 250mL corresponded to the maximum joint sensitivity and specificity on the ROC plot (Figure 1). We identified 52 (37.4%) patients with a high IBL volume (≥250mL). Clinicopathological features of patients with low and high IBL volumes are presented in Table 2. Multivariate logistic regression analyses determined that BMI, CRLM number, and CRLM tumor size were significantly associated with high IBL.

**Table 2:** Related patient factors according to different IBL volumes.

	**IBL**		**Univariate**	**Multivariate**	
	**≤250mL(n=87**)	**>250mL(n=52**)	***P***	**HR (95% CI)**	***P***
**Age**					
≤60 years	48	32	0.46		
>60 years	39	20			
**Gender**					
Male	55	36	0.47		
Female	32	16			
**BMI**					
≤25.3	74	31	<0.01*	4.3 (1.6–11.2)	<0.01*
>25.3	10	21			
**DM**					
Yes	10	4	0.50		
No	77	47			
**Hypertension**					
Yes	22	8	0.17		
No	65	44			
**HBV infection**					
Yes	48	32	0.46		
No	39	20			
**Number of CRLM**					
solitary	55	18	<0.01*	2.7 (1.1–6.5)	0.03*
multiple	32	34			
**Tumor size of CRLM (cm)**					
≤4.5	74	29	<0.01*	4.7 (1.9–12.0)	<0.01*
>4.5	12	22			
**Preoperative chemotherapy**					
Yes	34	33	<0.01*	1.8 (0.7–4.3)	0.22
No	53	19			
**Simultaneous primary and liver resection**					
Yes	42	17	0.07		
No	45	35			
**Type of hepatectomy**					
Anatomy	9	8	0.38		
Nonanatomy	78	44			

* Statistically significant (*P* <0.05)

### Effects of each predictor on long term survival

**Figure 2:**
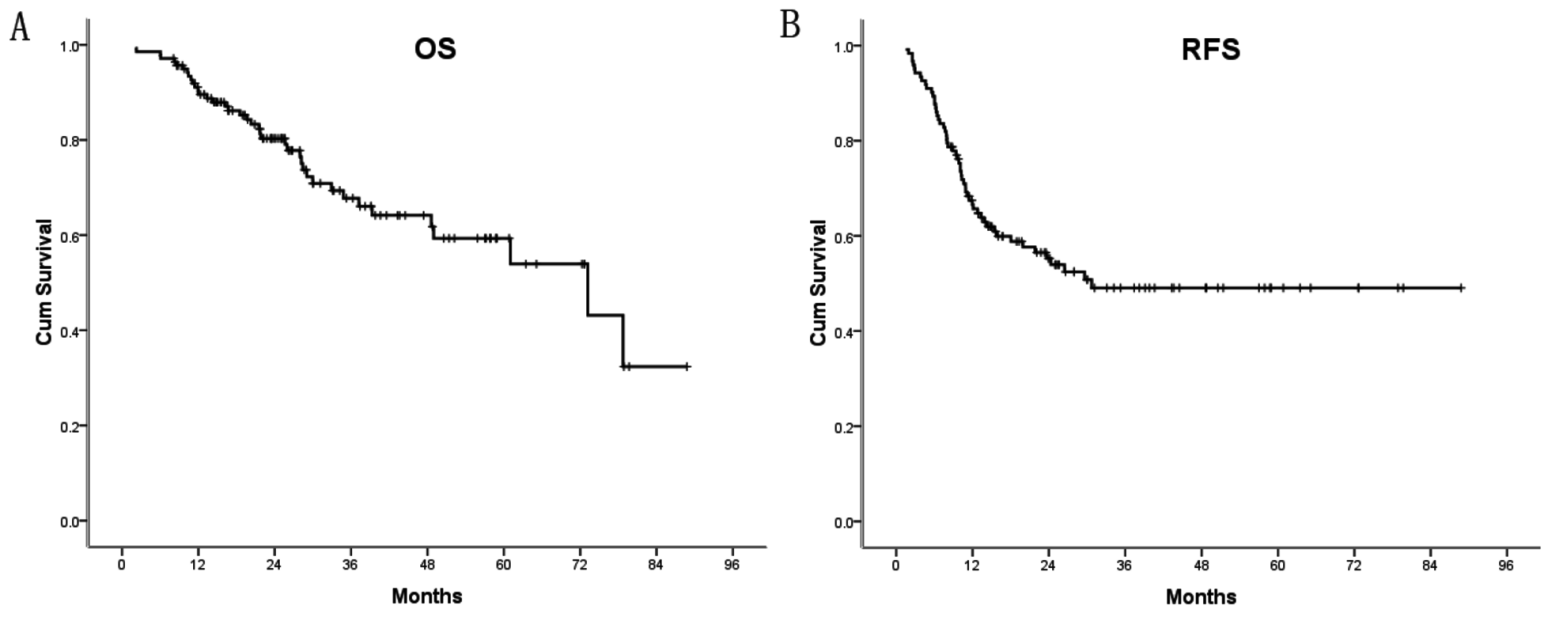
Kaplan–Meier curves for overall survival (a) and recurrence free survival (b) after CRLM resection for the entire patient cohort. The median OS for all patients was 73.2 months, and the proportion of patients alive at 3 and 5 years was 65% and 53%, respectively. The median RFS period was 30.7 months.

All 139 patients were followed up until December 2012. The median follow up period was 25 months (range, 2.1–88.8 months), the median OS for the entire group was 73.2 months, and the proportion of patients alive at 3 and 5 years was 65% and 53%, respectively (Figure 2a). We performed the Kaplan–Meier analyses to identify factors associated with OS after hepatectomy for CRLM. Eight factors, including BMI, LDLC/HDLC, lymph node metastasis of the primary tumor, CRLM tumor size, IBL, perioperative transfusion, and preoperative CEA and CA19-9 levels revealed significant correlation with long term survival through univariate analysis. However, only BMI (P=0.01; HR = 3.3; and 95% CI = 1.3–8.5) and IBL (P<0.01; HR = 4.5; 95% CI = 1.6–12.5) were independent predictors of OS through multivariate Cox regression analysis (Table 3).

**Table 3:** Cox-regression results regarding overall survival in liver resection for CRLM.

	**Univariate**		**Multivariate**	
	**HR(CI)**	***P***	**HR(CI)**	***P***
**Age**				
≤60years	1	0.12		
>60years	0.6 (0.3–1.1)			
**Gender**				
Male	1	0.39		
Female	0.7 (0.4–1.5)			
**BMI**				
≤25.3	1	<0.01*	1	0.01*
>25.3	2.7 (1.3–5.4)		3.3 (1.3–8.5)	
**DM**				
Yes	1	0.10		
No	2.0 (0.9–4.9)			
**NLR**				
≤1.9	1	0.06		
>1.9	1.9 (0.9–3.8)			
**LDLC/HDLC**				
≤3.7	1	0.03*	1	0.72
>3.7	2.1 (1.1–4.2)		1.2 (0.5–3.1)	
**HBV infection**				
Yes	1	0.92		
No	1.0 (0.5–1.8)			
**Primary tumor**				
Colon	1	0.86		
Rectum	1.1 (0.6–2.0)			
**LN metastasis of primary tumor**				
N_0_	1	0.048*	1	0.11
N_1–2_	2.3 (1.0–5.3)		2.2 (0.8–5.8)	
**Number of CRLM**				
Solitary	1	0.09		
Multiple	1.7 (0.9–3.2)			
**Size of CRLM (cm)**				
≤4.5	1	<0.01*	1	0.19
>4.5	3.6 (1.9–7.0)		1.9 (0.7–4.9)	
**Exhepatic metastasis**				
Yes	1	0.39		
No	1.5 (0.6–3.9)			
**Preoperative chemotherapy**				
Yes	1	0.56		
No	1.2 (0.6–2.3)			
**Type of hepatectomy**				
Anatomy	1	0.07		
Nonanatomy	0.5 (0.2–1.1)			
**IBL(mL)**				
≤250	1	<0.01*	1	<0.01*
>250	3.9 (2.0–7.6)		4.5 (1.6–12.5)	
**Perioperative transfusion**				
Yes	1	<0.01*	1	0.49
No	2.8 (1.4–5.3)		1.4 (0.6–3.5)	
**Perioperative morbidity**				
Yes	1	0.25		
No	1.7 (0.7–4.0)			
**Preoperative CEA (ng/mL)**				
≤20	1	0.02*	1	0.18
>20	2.1 (1.1–4.0)		1.8 (0.8–4.5)	
**Preoperative CA19-9 (U/mL)**				
≤35	1	0.01*	1	0.09
>35	2.3 (1.2–4.3)		2.2 (0.9–5.4)	
**Preoperative CRP (ng/L)**				
≤8.2	1	0.49		
>8.2	1.3 (0.6–3.1)			

* Statistically significant (*P* <0.05).

### Effects of each predictor on tumor recurrence

After CRLM resection, 63 (45.3%) patients experienced tumor recurrence. The median RFS period was 30.7 months (Figure 2b). According to the Cox proportional hazards model, NLR (*P*<0.01; HR = 2.8; 95% CI =1.4–5.6), LDLC/HDLC levels (*P*=0.03; HR = 2.4; 95% CI =1.1–5.2), tumor size of CRLM (*P*=0.04; HR = 2.1; 95% CI =1.0–4.2), IBL(*P*=0.02; HR = 2.2; 95% CI =1.1–4.1), and preoperative CRP levels (*P*=0.04; HR = 3.7; 95% CI =1.1–12.8) were significantly associated with poorer RFS (Table 4).

**Table 4:** Cox-regression results regarding recurrence-free survival in liver resection for CRLM.

	**Univariate**		**Multivariate**	
	**HR(CI)**	***P***	**HR(CI)**	***P***
**Age**				
≤60years	1	0.06		
>60years	0.6 (0.3–1.0)			
**Gender**				
Male	1	0.46		
Female	0.8 (0.5–1.4)			
**BMI**				
≤25.3	1	0.04*	1	0.32
>25.3	1.8 (1.0–3.4)		0.7 (0.3–1.4)	
**DM**				
Yes	1	0.45		
No	0.6 (0.2–2.1)			
**NLR**				
≤1.9	1	<0.01*	1	<0.01*
>1.9	2.5 (1.4–4.4)		2.8 (1.4–5.6)	
**LDLC/HDLC**				
≤3.7	1	0.02*	1	0.03*
>3.7	2.0 (1.1–3.6)		2.4 (1.1–5.2)	
**HBV infection**				
Yes	1	0.71		
No	1.1 (0.7–1.9)			
**Primary tumor**				
Colon	1	0.29		
Rectum	1.3 (0.8–2.3)			
**LN metastasis of primary tumor**				
N_0_	1	0.13		
N_1–2_	1.6 (0.9–3.1)			
**Number of CRLM**				
Solitary	1	<0.01*	1	0.08
Multiple	2.5 (1.5–4.4)		2.0 (0.9–4.3)	
**Size of CRLM (cm)**				
≤4.5	1	<0.01*	1	0.04*
>4.5	3.4 (2.0–5.9)		2.1 (1.0–4.2)	
**Exhepatic metastasis**				
Yes	1	0.06		
No	2.3 (0.9–5.3)			
**Preoperative chemotherapy**				
Yes	1	<0.01*	1	0.20
No	3.1 (1.8–5.5)		1.7 (0.8–3.5)	
**Type of hepatectomy**				
Anatomy	1	0.49		
Nonanatomy	0.8 (0.3–1.7)			
**IBL(mL)**				
≤250	1	<0.01*	1	0.02*
>250	3.2 (1.8–5.2)		2.2 (1.1–4.1)	
**Perioperative transfusion**				
Yes	1	0.02*	1	0.32
No	1.9 (1.1–3.5)		0.7 (0.4–1.4)	
**Perioperative mortality**				
Yes	1	0.85		
No	1.1 (0.5–2.5)			
**Preoperative CEA (ng/mL)**				
≤20	1	0.92		
>20	1.0 (0.6–1.9)			
**Preoperative CA19-9 (U/mL)**				
≤35	1	0.98		
>35	1.0 (0.6–1.8)			
**Preoperative CRP (ng/L)**				
≤8.2	1	0.04*	1	0.04*
>8.2	0.3 (0.1–1.0)		0.3 (0.1–0.9)	

* Statistically significant (*P* <0.05).

### Correlation between IBL, survival, and recurrence

**Figure 3:**
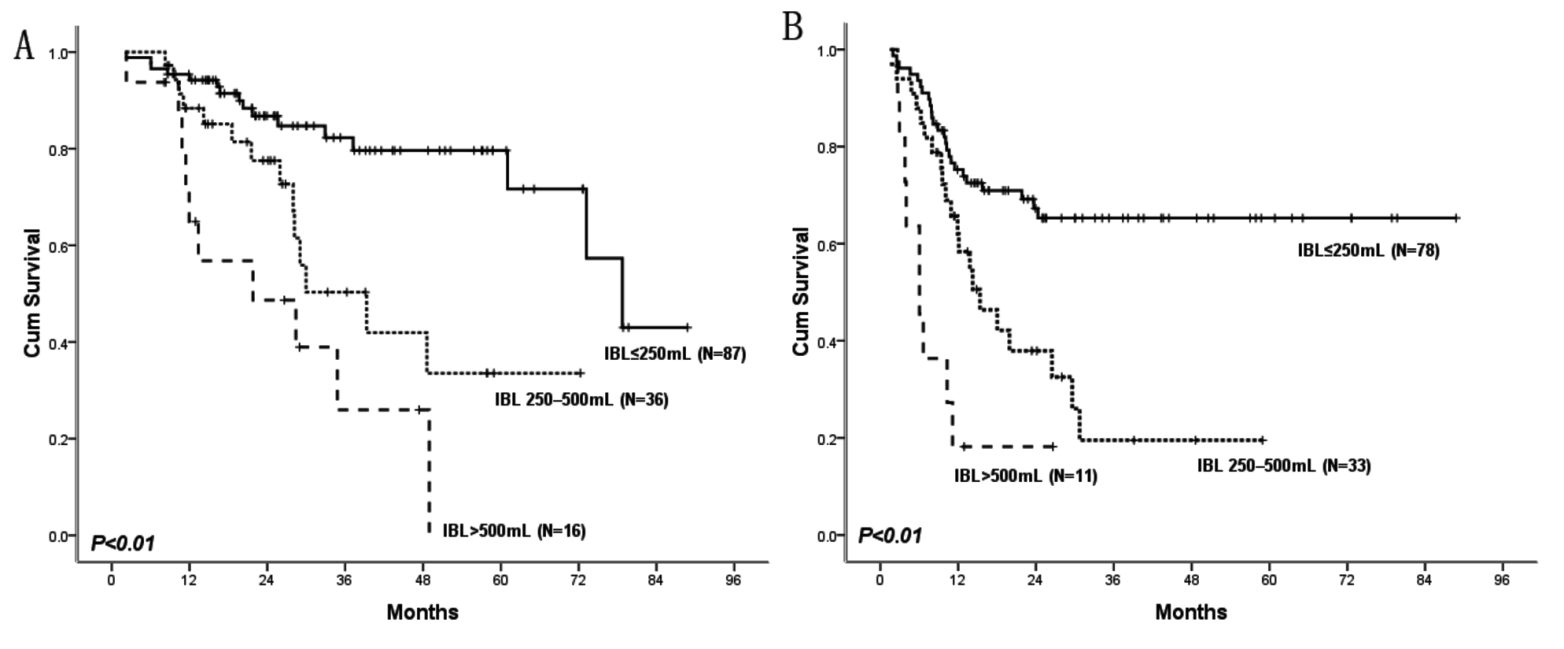
Dose–response relationship between IBL, OS (a), and RFS (b). OS and RFS decreased significantly with increasing IBL volumes. The 5-year OS of patients with IBL≤250mL, 250–500mL, and >500mL were 71%, 33%, and 0%, respectively (*P*<0.01). RFS of patients within the three IBL volumes at the end of the first year were 67%, 38%, and 18%, respectively (*P*<0.01).

Because IBL was an independent predictor of long term survival and tumor recurrence after CRLM resection, we doubted whether there was a correlation between the degree of IBL and duration of OS and RFS. We stratified our patients into 3 groups based on the degree of IBL and observed that the OS and RFS periods significantly decreased with increasing IBL volumes(Figure 3). The 5-year OS rate of patients with IBL≤250mL, 250–500mL, and >500mL were 71%, 33%, and 0%, respectively (*P*<0.01). Moreover, the RFS rates of patients within the three IBL levels at the end of the first year were 67%, 38%, and 18%, respectively (*P*<0.01).

## Discussion

Growing evidence has suggested that hepatic resection is the only current potentially curative therapy against liver metastases of colorectal origin [13,14]. However, the high postoperative recurrence rate, which up to 50%–70% [15], restricts further patient benefit. Although numerous studies have concentrated on prognostic factors of surgical resection for CRLM [2,16,17], most of the factors were associated with underlying conditions or tumor characteristics, and the role of perioperative management is rarely investigated.

Several recent studies have reported the prognostic role of IBL after HCC resection. For example, Katzet al[10]. reviewed 192 patients who underwent partial hepatectomy for HCC and revealed that patients with an IBL volume >1L exhibited significantly poor long term outcomes and high recurrence rates. Moreover, they reported a notable negative correlation between increasing IBL volumes and length of disease-specific survival. In addition, Chikamoto et al. [17] reported consistent results in patients without blood transfusion. Nevertheless, studies focusing on CRLM are uncommon. Our research is the first to demonstrate the impact of IBL on long term outcome and tumor recurrence, and our results indicated that treatment efficacy for advanced colorectal cancer was also dependent on the surgeons’ experience with delicate procedures.

The IBL volume was closely related to the patient’s physical state and tumor stage. In the present study, BMI, CRLM number, and CRLM tumor size were observed to be predictors of high IBL volumes through multivariate analyses. The thick abdominal wall and abundant soft tissues associated with high BMI can significantly limit surgical exposure and make the surgical procedure more difficult [18]. CRLM number and tumor size indicate advanced tumor progression and a greater number of surgical procedures. According to the ROC curves, we determined 250mL as the cutoff value for low and high IBL volumes in our study, which was much less than that reported in previous studies focusing on HCC resection. This could probably be attributed to the strict patient selection in our cohort. In stage IV patients, those with high risk for perioperative complications such as unfavorable tumor location or poor liver function were recommended to systemic chemotherapy instead of surgery.

Previous reports have suggested that blood transfusion, which is closely related to IBL, was an independent predictor of survival and recurrence in colorectal cancer [19-21]. However, not all of these studies included IBL in their analyses; thus, the association of transfusion with poor oncological outcome might be partly because of unmeasured effects of IBL. Harlaar et al. [22] designed a randomized controlled trial to evaluate the effects of allogeneic blood transfusions and an autologous blood transfusion program in patients with colorectal cancer; they reported that after a 20-year follow up period, the overall and colorectal cancer-specific survival rates were worse in patients in the autologous transfusion group, but no differences were observed in survival between the groups receiving transfusions. Thus, he speculated that the adverse effects were the result of a combination of blood donation before surgery and IBL in the autologous transfusion group. In our study, blood transfusion indicated predictive significance for survival and recurrence after CRLM resection only by univariate analysis. In contrast, IBL was a significant predictor of outcome by both univariate and multiple analyses. Furthermore, we evaluated different IBL volumes and demonstrated a dose–response relationship between increasing IBL volumes and long term survival and tumor recurrence.

Although IBL seems to be a sensitive indicator of treatment outcome, potential explanations remain unclear. First, IBL might increase the risk for intraoperative tumor spillage and hematogenous spread, which could result in recurrence [10]. Furthermore, large amounts of blood loss can induce tissue hypoperfusion and inadequate oxygenation. These events depress mitogen-induced lymphocyte proliferation [22] and interleukin-2 production [23], thereby impeding antitumor immunity, and also up regulate vascular endothelial growth factor, thereby accelerating tumor angiogenesis [24]. In addition, hypoxia promotes genomic instability and leads to a variety of genetic changes, which results in the development of a more aggressive phenotype of the residual tumor cells [25].

There were a few limitations to this study. First, it was retrospective in nature and the possibility of recall bias should be considered. Second, the degree of IBL was not a precise measurement but was rather estimated by the surgeon and the circuit nurse; thus, over and/or under estimation of IBL was possible. A difference between preoperative and postoperative hemoglobin levels might provide more accurate data [18]. However, hemoglobin assessment is not routinely performed in our department; therefore, data regarding differences in hemoglobin levels is limited. Moreover, there were 59 (42.4%) patients who underwent simultaneous resection for the primary lesions as well as liver metastases. Although no significant relationship was observed between simultaneous resection and IBL, further studies limited to hepatectomy for CRLM are warranted.

In conclusion, we determined that IBL during CRLM resection is an independent predictor of long term survival and tumor recurrence. The prognostic value of IBL was confirmed by the dose–response relationship, in which a greater IBL volume was associated with a higher recurrence rate and poorer long term survival. These findings highlight the importance of selecting optimal patients and also alert surgeons to take precautionary measures to minimize IBL.
